# West Nile Virus Aseptic Meningitis and Stuttering in Woman

**DOI:** 10.3201/eid1708.101691

**Published:** 2011-08

**Authors:** Nardeen Mickail, Natalie C. Klein, Burke A. Cunha

**Affiliations:** Author affiliations: Winthrop-University Hospital, Mineola, New York, USA; and State University of New York School of Medicine, Stony Brook, New York, USA

**Keywords:** West Nile virus, viruses, aseptic meningitis, stuttering, letter

**To the Editor:** West Nile virus (WNV), a mosquito-borne flavivirus, is closely related to St. Louis encephalitis virus and Japanese encephalitis virus (JEV). Most cases of WNV have been mild, but neuroinvasive disease has been observed, especially among older persons and immunocompromised persons (*1**,**2*). The most common neurologic manifestations of WNV are aseptic meningitis, meningoencephalitis, and encephalitis with or without acute flaccid paralysis (*3*). Other less common neurologic manifestations include Guillain-Barré syndrome, chorioretinitis, stroke-like symptoms, and unilateral brachial plexopathy (*4**,**5*).

We report a case of WNV aseptic meningitis in a 39-year-old immunocompetent woman who had severe headache with new-onset stuttering. Her medical history included lumbar disc herniation and migraines, for which she was taking sumatriptan. Her symptoms started ≈2 weeks before hospitalization and included a severe generalized headache initially thought to be a migraine, but sumatriptan resulted in no improvement. A few days later, she had fever and was intermittently stuttering. She denied recent travel or animal exposure but admitted to having received multiple mosquito bites during the preceding weeks.

At admission, she had a temperature of 101.3°F, pulse rate of 92 beats/min, blood pressure of 130/80 mm Hg, and respiratory rate of 16 breaths/min. She appeared mildly ill but was alert and oriented with no nuchal rigidity, photophobia, rash, or limb weakness. Results of a physical examination were unremarkable, and results of a neurologic examination were notable only for stuttering. Laboratory test results included a leukocyte count of 12,300 cells/mm^3^ (63% neutrophils, 29% lymphocytes, 7% monocytes, 1% basophils) and a platelet count of 204,000 cells/mm^3^. Other laboratory values were unremarkable, and levels of serum transaminases and creatinine phosphokinase were within reference ranges. Cerebrospinal fluid (CSF) was clear and contained 37 leukocytes/mm^3^ (2% neutrophils, 78% lymphocytes, 20% monocytes), 2 erythrocytes/mm^3^, a glucose level of 68 mg/dL, a protein level of 36 mg/dL, and a lactic acid level of 2.1 meq/L. No abnormalities were found on a cranial computed tomography scan.

The patient began treatment with acyclovir, 10 mg/kg intravenously, every 8 hours for 3 days. On hospital day 2, she underwent magnetic resonance imaging of the brain; results were within reference limits. On hospital day 3, her headache began to improve and she became afebrile, but she still stuttered occasionally. Results of CSF tests for enterovirus, herpes simplex viruses 1 and 2, and varicella zoster virus and PCR for human herpesvirus 6 were negative, and acyclovir was discontinued. On hospital day 5, she was discharged. Three days later, serum and CSF ELISA results for WNV were positive. A WNV ELISA was performed at ViroMed Laboratories (Minnetonka, MN, USA) by using a Focus Test Kit (Focus Diagnostics, Cypress, CA, USA), and the result was positive. The patient subsequently reported that her stuttering had ceased.

A high degree of clinical suspicion for WNV infection should be considered in patients with a recent history of mosquito bites and an acute febrile illness associated with neurologic signs and symptoms (*5*). Typical CSF findings of infection with WNV include lymphocytic pleocytosis, elevated protein level, reference glucose and lactic acid levels, and no erythrocytes (*6*).

The clinical presentation of WNV infection varied widely from asymptomatic seroconversion to fatal encephalitis. It is possible, but unlikely, that the stuttering in the patient was an indication of a migraine aura. Initially, the patient reported that the headache might have been a migraine, but later reported that its associated symptoms, e.g., photophobia, were not as severe and did not last as long as her usual migraines. Further argument against migraine aura is the lack of response to her migraine medication and the fact that the stuttering continued after the headache resolved.

Because WNV resembles JEV, it is interesting to note that a case of stuttering in a young adult infected with JEV has been reported (*7*). However, the mechanism of stuttering associated with WNV is unknown. One possible explanation is myoclonic contractions of the tongue, i.e., vocal myoclonus.
